# Spirituality issues in cancer patients at Ocean Road Cancer Institute, Dar es Salaam, Tanzania

**DOI:** 10.1017/S1478951525101144

**Published:** 2025-12-15

**Authors:** Veronica Bernard Mkusa, Nazima Dharsee, Janepher Nyakake, Stanley Wilson Acuda

**Affiliations:** 1 Education and Research, Palliative Care Trainer and Researchers Network of Tanzania: Master of Science in Palliative Care Graduate, IHPCA/Makerere University,Dar es Salaam, Tanzania; 2 Education and Research, Ocean Road Cancer Institute, Dar es Salaam, Tanzania; 3 Education and Research, Institute of Hospice and Palliative Care in Africa/Makerere University, Kampala, Uganda

**Keywords:** Palliative care, spirituality issues, cancer patients, Ocean Road Cancer Institute, Tanzania

## Abstract

**Objectives:**

To explore the spirituality issues among cancer patients undergoing cancer treatment at Ocean Road Cancer Institute (ORCI), Dar es Salaam, Tanzania.

**Methods:**

This was a qualitative study involving purposively selected cancer patients receiving treatment at ORCI, Dar es Salaam, Tanzania. Data were collected through one-to-one audiotaped interviews using a pilot-tested semi-structured interview guide. Data saturation was reached at the 24th interview. Thematic data analysis was used.

**Results:**

Twenty-four cancer patients attending ORCI, consisting of 12 males and 12 females, aged 18–65, participated in the study. The majority of participants, 71% (*n* = 17), had low education, 71% (*n* = 17) were of low socioeconomic status, and 83% (*n* = 20) were either Christians or Muslims. Six broad themes emerged: aspects of life contributing to meaning and purpose of life, beliefs surrounding cancer, effects of cancer on spirituality, spirituality in relation to seeking health care, spirituality and coping with cancer, and spirituality needs of cancer patients.

**Significance of results:**

Cancer patients at ORCI face significant spirituality issues and hold misconceptions about the causes and treatment of cancer. Palliative care practitioners must routinely identify and address spiritual issues of cancer patients in order to improve the quality of life of cancer patients.

## Introduction

Spirituality is a sense of feeling that an individual has that there is someone greater than humankind. It is the aspect of humanity that refers to the way individuals seek and express meaning and purpose, and the way they experience their connectedness to the moment, to self, to others, to nature, and to the significant or sacred. It is an essential element that enables a person to make meaning in life. It can make a person ask themselves the following questions: why me? What is happening to me, and what will happen now? What does it mean? (Puchalski 2012a, 2012b). Spirituality and existential issues are important since many patients experience these issues while coping with chronic, potentially fatal illnesses like cancer (Ferrell et al. 2018).

Spiritual care is significant in palliative care, as patients’ perspectives and religious demands may change during terminal disease, necessitating a holistic approach from practitioners (Richardson 2014). Faith significantly impacts palliative care, particularly for individuals with chronic and life-threatening conditions, by enhancing their quality of life (Miller et al. 2024). Spirituality is a vital and essential part of humanity. Depending on their spiritual practices and beliefs, patients with advanced cancer may feel less pain and distress and have a higher quality of life (Winkelman et al. 2011).

Spirituality provides motivation, self-esteem, and a sense of purpose, enabling individuals with life-limiting illnesses to cope with challenges and maintain daily activities without complaint (Barber and Wilson 2015). An earlier study done in Tanzania reported that spiritual care was primarily viewed as a religious practice, requiring the incorporation of religious beliefs into nursing care to meet patients’ spiritual needs (Dhamani et al. 2011). Spirituality significantly improves patients’ quality of life and is crucial for providing high-quality palliative care. Thus, emphasizing holistic care and quality of life (Lee 2019).

Holistic care includes providing patients with chronic illnesses, serious illnesses, and life-threatening illnesses with physical, psychological, social, and spiritual care (Speck 2016). Palliative care practitioners should be aware that listening to patients’ stories can be therapeutic, despite not having the ability to solve all patients’ issues.

Addressing spirituality is crucial for patients’ quality of life, as reported by Puchalski et al. (2012a) that a strong spiritual foundation can significantly enhance patients’ well-being and that spirituality is a crucial component of a cancer patient’s care from the time of initial diagnosis, throughout the course of their sickness and death (Puchalski 2012b).

A person diagnosed with cancer may experience a great deal of worry as a result of the diagnosis, including fear of the condition, fear of tests, and fear of treatment. Anxiety, sadness, and feelings of hopelessness stem from fear of the future (PeaceHealth and Bill 2023). A cancer diagnosis can lead to anxiety, concerns about the future, treatment repercussions, and social problems, including financial difficulties, as the individual may struggle to continue working (Murphy et al. 2018). One of the 8 recognized domains of quality palliative care is spirituality. Understanding the connection between a patient’s spirituality and their religion and existential issues is therefore important since many patients experience these issues while coping with chronic or potentially fatal illnesses like cancer (Ferrell et al. 2018).

Modern healthcare services often prioritize patient healing over spirituality. However, it is crucial to address patients’ spiritual needs in order to provide a comprehensive package of care that will improve patients’ quality of life (Forouzi et al. 2017). This statement is supported by a recent study in the United States and Switzerland that gathered nurses’ opinions on how to address the spiritual needs of cancer patients (Zumstein-Shaha et al. 2020). The study revealed that nurses providing spiritual care to cancer patients effectively helped the patients find meaning in life and improved their coping mechanisms. Several other studies have demonstrated the advantages of addressing cancer patients’ spiritual needs. For instance, a meta-analysis of studies on spiritual therapies for cancer patients by Xing et al. (2018) found that spirituality significantly enhanced patients’ quality of life by addressing feelings of depression, anxiety, and hopelessness. Additionally, a study conducted in Poland on Catholic cancer patients found that the patients’ spirituality and religiosity affected their quality of life in terms of their ability to function physically, mentally, emotionally, and socially (Majda et al. 2022).

Based on the above reports, it is essential that palliative care for cancer patients should not only focus on physical symptoms but it should also address their psychological, social, and spiritual needs. Importantly, it should be noted that apart from a single study on nurses’ understanding of spirituality by Dhamani et al. (2011), no previous study had been done in Tanzania that focuses on spiritual issues among cancer patients.

ORCI attends to about 10,000 new patients and 55,000 follow up patients per year. This study therefore aimed to provide evidence-based understanding of spiritual issues and make recommendations for improvement of spiritual care for cancer patients at Ocean Road Cancer Institute (ORCI).

## Materials and methods

The Consolidated Criteria for Reporting Qualitative Studies (COREQ) was used to frame the reporting of results.

### Study design

A qualitative study was used to explore the spirituality issues of cancer patients at ORCI.

### Study setting

The study was conducted at the ORCI in Dar es Salaam, Tanzania. ORCI is the national cancer referral center that treats over 50,000 patients annually, including over 28,000 cancer patients, from all over the country and neighboring countries. Services provided at the Institute include cancer treatment, early detection, prevention through various screening services and vaccination, research, teaching, and palliative care (ORCI 2025).

### Participants

Participants were purposively selected from among inpatient and outpatient departments of the Institute. Inclusion criteria: All patients 18 years and above who were receiving cancer treatment at the Institute, who were deemed by their physicians to be medically fit to be interviewed, and who were willing to give informed consent to participate in the study.

Exclusion criteria: The study excluded all patients under 18 years who were deemed medically unfit or unwilling to give informed consent to participate in the study.

### Data collection methods and tools

#### Methodological rigor

The study used a Kiswahili interview guide to interview purposively selected Kiswahili-speaking patients who were receiving cancer treatment at ORCI. Kiswahili is the most widely spoken language in Tanzania, and all participants in this study were fluent in Kiswahili. The interviews were conducted face-to-face by VM, the Principal Investigator, in private rooms, and confidentiality was guaranteed. Each participant was given and read the information leaflet about the study and signed the informed consent form. All interviews were recorded.

Thematic saturation, the point at which no new information emerged from the interviews, was reached at the 24th interview. Duration of each interview ranged from 20 to 30 minutes, and data collection was completed in 4 weeks. The reliability and validity of the data were assessed by comparing the results from in-depth interviews with those from a focus group discussion involving 5 cancer patients (3 females and 2 males), employing triangulation as a methodological approach (Guion L et al. 2011).

Transcription was done after the interviews, and translation into English was done by 2 coders who were not part of the authors. Then, back-translation to Kiswahili was done to ensure trustworthiness. One (1) focus group discussion was conducted involving 5 cancer patients.

The interview guide was self-designed. It was pretested on 3 cancer patients who were not included in the study, and some modifications were made.

The interview guide had 3 main questions:

- What gives your life meaning?
- Has the cancer illness made any impact on your relationship with God?
- What makes you be able to cope with the cancer illness you have?

#### Sampling process

The senior nurses in charge of in-patient wards and out-patient clinics identified eligible participants to be interviewed. All patients who were identified and requested to participate agreed, and there were no dropouts.

## Data analysis

Content analysis was used to examine the transcription. This was done by reading the transcription first to get familiar with it. NVIVO software version 12 (Lumivero 2021) was used to analyze the data. Then, coding was done, the coding process facilitated a process of establishing meaning from the data and putting together the flow of evident information. Subthemes were created by grouping together comparable codes; these subthemes were then further categorized based on similarity, and themes were created.

## Results/findings

### Demographic characteristics of study participants

A third of the study participants, 33% (*n* = 8), were in the age category of 45–54 years, and only 13% (*n* = 3) were aged 65 years and above. Half of participants, 50% (*n* = 12), were females, and the other half, 50% (*n* = 12), were males. About half of the participants did not attend school at all, 13% (*n* = 3), or did not complete primary education, 42% (*n* = 10). Only a few completed secondary school, 17% (*n* = 4), or attended college 8% (*n* = 2). More than half of participants, 54% (*n* = 13), were married. Occupation-wise, 38% (*n* = 9) of the participants were peasant farmers, and others were engaged in small businesses 33% (*n* = 8). The majority of the participants were either Christians, 42% (*n* = 10), or Muslims, 42% (*n* = 10), and a few, 17% (*n* = 4), had no religion.

## Findings

Six broad themes and 16 subthemes emerged from the interviews (Table 2). The themes are: Aspects of life contributing to the meaning and purpose of life during cancer illness; Beliefs surrounding cancer disease; Effect of cancer on spirituality of the patients; Spirituality in relation to seeking health care; Spirituality in relation to coping with cancer illness, and spirituality needs of the cancer patients.

**Table 1.** Demographic characteristics of study participants

Characteristic of Participants	Number/percentage
Education level	
Never attended school	3 (13%)
Did not complete primary school	7 (29%)
Primary school	7 (29%)
Secondary school	4 (17%)
College	2 (8%)
Others (adult education)	1 (4%)
Total	**24 (100%)**
Marital status	
Married	13 (54%)
Single	5 (21%)
Separated	4 (17%)
Widow/widower	1 (4%)
Cohabiting	1 (4%)
Total	**24 (100%)**
Occupation	
Peasant farmer	9 (38%)
Small business	8 (33%)
Salaried worker	1 (4%)
Others	6 (25%)
Total	**24 (100%)**
Religion	
Muslim	10 (42%)
Christian	10 (42%)
No religion	4 (17%)
Total	**24 (100%)**

**Table 2.** Themes and sub-themes

Themes	Sub-themes
Theme 1: Aspects of life that give meaning and purpose during cancer illness	Faith /believing in God
	Life/well-being
Theme 2: Beliefs surrounding cancer disease and spirituality of a patient	Misconception/superstitious beliefs/Bewitched beliefs
	Modern cancer treatment kills quickly
Theme 3: Effect of cancer disease on spirituality of the cancer patients	Faith in recovering and being cured of the illness
	Effect of cancer on how patients value themselves
	Strengthened faith in God
	Financial constraint
	Abandonment by spouse
Theme 4: Spirituality of patients in relation to seeking health care	Belief that God and hospital care are both important
	Delay in seeking health care due to beliefs in witchcraft
Theme 5: Spirituality of patients and coping with cancer illness	Faith in God and healing
	Acceptance of the cancer disease
Theme 6: Spirituality needs of cancer patients	Prayers to get healing from God
	Good relationship with family
	Health care workers to understand and care for us

### Theme 1: Aspects of life that give meaning and purpose of life during cancer illness

The majority (92%, *n* = 22) of participants identified faith, good health, work, and well-being as important elements in life during cancer illness, prompting them to reflect on their purpose and meaning.

*The first thing that gives me peace is to believe in God, that he is powerful, He is everything and what is happening to me and what I am going through right now.* (IDI, Respondent 1, Outpatient).

*What matters to my life first is health, when you are healthy in your life then you know who this is and what this is.* (IDI, Respondent 11, Outpatient)

Spirituality provides purpose, motivation, and self-esteem, enabling individuals to persevere despite challenges, and faith encourages them to carry out daily activities without complaint.

### Theme 2: Beliefs surrounding cancer disease and spirituality

The study revealed that participants held diverse beliefs about cancer, with 67% (*n* = 16) believing in God and believing cancer is like any other disease despite family influences, and 33% (*n* = 8) initially believing it’s a bewitched disease but later changed their views during the course of illness.

*;When someone gets sick they usually go through many ways including traditional healers. So, I was taken through such ways when I saw that there was no help, I decided to stay in the hospital and pray to God believing that anything is possible and anything can happen if I serve God along with the medicines that I take.* (IDI, Respondent 24, Inpatient).

Some participants 29% (*n* = 7) believed modern cancer treatment kills quickly, so they preferred local herbs over hospital treatment, as they had heard that many patients receiving hospital treatment died without healing.

*My family does not have faith and is afraid of this treatment. We have experience with patients who went through this treatment and died, so they feel that this treatment kills quickly. So, my family is afraid. but so far, I am stable and I didn’t want to listen to street doctors. It is me, my God and the hospital.* (IDI, Respondent 4, Outpatient)

### Theme 3: Effect of cancer diagnosis on spirituality of the participants

The study found that cancer diagnosis can impact spirituality, self-value, finances, and marriage, with 63% (*n* = 15) of participants hoping for recovery and others relying on God.

*I have faith in God, I thank Him every time that is why I came here.* (IDI, Respondent 13, In-patient)

Some participants felt that their self-worth was negatively impacted by physical deformity or inability to work, while others 21% (*n* = 5) had to sell their assets to meet treatment costs, which exacerbated their financial problems.

*Honestly, it has actually affected my self-esteem, I feel like my self-worth has decreased.* (IDI, Respondent 22, Inpatient)

*Life is going downhill, you’re selling your things to find money for treatment so you can survive, eh.* (IDI, Respondent 21, In-patient)

The study found that 17% (*n* = 4) of participants with cancer experienced abandonment by their spouses, which worsened their pain and prompted them to seek help.

*…my wife told my family members that she could not live with a patient who can’t even take a shower. She left me with the children, one eight years old another three years old.* (IDI, Respondent 6, Inpatient).

### Theme 4: Spirituality of patients in relation to seeking health care

The study revealed that 33% (*n* = 8) of participants initially sought spiritual help from traditional healers, 25% (*n* = 6) sought medical care due to belief in hospital care and faith in God, and only 8% (*n* = 2) sought health care first.

*…when you get sick go to the hospital, religion is there because it is not a be-witched disease, it is a hospital disease and a hospital treatment, so if you go to the hospital then religion follows.* (IDI, Respondent 16, In-patient)

Other participants 33% (*n* = 8) who had spirituality-related concerns delayed seeking medical attention because they believed in witchcraft.

One such participant said:

*Initially, the family believed that I was bewitched, that statement compelled me to live and seek health services at Muhimbili Hospital. After undergoing tests, I was diagnosed with cancer therefore I refuted the statement that I was bewitched.* (IDI, Respondent 8, Outpatient)

### Theme 5. Spirituality and coping with cancer

Most respondents, 83% (*n* = 20), used spirituality as a coping strategy for their cancer diagnosis, demonstrating resilience to endure the disease.

*I do what my soul believes, I believe God is my father, should I be treated this way or that way, I have faith in him*. (IDI, Respondent 19, In-patient)

Similarly, belief in faith as a coping strategy was reported in a focus group discussion which also emphasized the significance/importance of family support

*Faith has made me stand. When I was told the results of the test I was shocked but then through faith I remained stable, so I told my children and we came all the way to ORCI in Dar es Salaam for treatment and my children got stronger when they saw that I had a strong faith.* (FGD, Respondent 6, Outpatient)

### Theme 6. Spirituality needs of cancer patients

This study revealed the participants’ spiritual needs, including prayers, seeking God’s healing, maintaining good family relationships, and healthcare workers’ understanding and care, which helped them feel calm, peaceful and closer to God.

*In my faith, they pray for me, this is my greatest need, even when I call, they pray for me so it really gives me comfort.* (FGD, Respondent 4, Outpatient).

## Discussion

This study aimed to explore spirituality issues in cancer patients at ORCI, Dar es Salaam, Tanzania. The sociodemographic characteristics of study participants, who were mostly peasant farmers or small business owners and had low education, and viewed faith in God as crucial for survival during cancer illness, regardless of their religion, gender, or socioeconomic status.

This finding differs from that of a study by Bottaro and Faraci (2022), which indicated considerable variation in sociodemographic characteristics in the coping techniques used by cancer patients. Women who were younger, in a relationship, with a high educational level, active working status, and a high salary had positive coping mechanisms.

Therefore, healthcare workers are advised to conduct spirituality assessments on patients with life-threatening or chronic illnesses and address individual spirituality needs regardless of age, sex, or religious orientation.

The study found that cancer patients’ faith in God was crucial for their resilience and perseverance during treatment, a finding that aligns with Barber’s (2022) Birmingham study.

Seeking health services helps individuals find meaning in life and well-being, and discussing treatment goals with the clinical and palliative care teams can help patients achieve their objectives. This was observed in a study where a cancer patient was involved in teaching medical students each semester about medical care from a patient’s point of view. She is nearing the end of her life, but is determined to continue giving those talks; she finds meaning in the huge impact she has made on future physicians. (Puchalski 2001). Therefore, health care workers must comprehend cancer patients’ thoughts and spirituality in order to effectively manage their conditions.

Regarding beliefs surrounding cancer and spirituality, the study has highlighted the importance of spirituality-related conversations for cancer patients, and challenges they may face, including superstitious beliefs, and emphasized the significance of spiritual growth.

This study has also indicated that family members’ cultural beliefs can lead to delayed medical attention, similar to Daher’s (2012) study, which found that cultural misconceptions in cancer patients can hinder surgery consent and result in disease progression. Several participants in this study initially believed that cancer was due to witchcraft but later realized that cancer was just a more advanced disease. Clinical and palliative care teams are therefore urged to improve patient education on cancer and its treatment.

This study found that patients with spiritual beliefs sought medical assistance and comfort through prayers and sacred texts, similar to findings of a study by Raffay (2022) and Jors et al (2015). Our study also found that spiritual leaders and believers often supported cancer patients, thus boosting their confidence and overall health and functioning, similar to findings of a study in Lebanon by Chaar et al. (2018), which reported that spirituality improved quality of life, enhanced cognition and emotions, and boosted cancer patients’ functioning.

About half of our participants, especially those with eye, breast, and nasopharyngeal cancers, and women unable to bear children, experienced lowered self-worth. This highlights the need for psychosocial support as reported in the study by Niveu and Beaudion (2021). Counseling and family support can help individuals with cancer cope with organ loss, but their spirituality requires understanding and support from the clinical and palliative care team and family.

In this study, some individuals with spiritual issues initially prioritized treatment from traditional healers over medical treatment but later sought medical attention, which implied that spirituality may have influenced their decision to seek medical help. Forouzi et al. (2017) had emphasized the importance of addressing spiritual issues in cancer patients to ensure prompt medical attention, prevent disease progression, and improve survival chances. Similarly, a study in Sub-Saharan Africa by Mwaka (20022) found that cultural beliefs significantly influenced cancer care, with patients seeking treatment from traditional healers or herbalists rather than from hospitals. These findings underscore the necessity for increased community education to raise awareness about cancer and its treatment in Tanzania.

ORCI patients in this study utilized spirituality as coping strategies, similar to results of studies from other developing countries, which reported positive impact of spirituality on the disease and its treatment (Gayatri et al. 2021). An earlier study in Tanzania had revealed that resilience among young cancer patients and children receiving cancer treatment was attributed to hope for healing and faith in God (Kohi et al. 2019). This study has highlighted individual patients’ unique coping mechanisms, which included faith in hospital services, acceptance of diagnosis, confidence in recovery, availability of medical care, and acceptance of treatments. Therefore, clinical and palliative care teams should constantly explore and support each patient’s unique coping strategy.

Finally, our study has identified the spiritual needs of cancer patients at ORCI, which include: prayers, seeking healing from God, maintaining healthy family relationships, and fostering compassionate healthcare from the Institute staff. These findings align with results of a study from Iran, which highlighted the importance of holistic care in addressing patients’ spiritual needs (Hatamipour et al. 2015). Therefore, addressing the spiritual needs of cancer patients can help manage their illness, promote strength, peace, and happiness despite their poor conditions (Moloko 2020).

## Study limitation

The study had a small sample size therefore, its results cannot be generalized to the very large number of cancer patients who attend ORCI and to the country at large. Nevertheless, saturation was utilized to determine sample size, which adds to the reliability of the findings. In addition, participants came from all over Tanzania and beyond, which can contribute to generalizability and representativeness of the findings. However, the study has pointed out how critical it is to address spirituality-related concerns of cancer patients, which may encourage other healthcare institutions to take spirituality issues of cancer patients into account in an effort to improve the quality of cancer care and quality of lives of cancer patients and their families.

## Conclusion

Cancer patients at ORCI face spirituality challenges, requiring prayers, family support, and care to maintain happiness, resilience, and overall health. Cancer diagnosis can significantly impact an individual’s spirituality, self-worth, finances, and marriage, and affects individuals of all ages, sexes, religions, marital statuses, education levels, and occupations. The study has revealed that spirituality influences individuals’ medical seeking behaviors, particularly cancer patients who often use spirituality as a coping mechanism for their illness.

Finally, the study has identified spiritual needs of cancer patients at ORCI, which include prayers, seeking healing from God, maintaining healthy family relationships, and fostering compassionate healthcare from staff.

## Recommendations

Clinical and palliative care teams should routinely explore, identify, and address spirituality issues in patients with life-limiting illnesses to enhance their quality of life. There is a dearth of research and publications on spirituality issues in Tanzania and in Sub-Saharan Africa. Therefore, there is an urgent need for more research on spirituality issues among patients, especially among those with life-threatening illnesses.

The following issues must be addressed in order to improve the quality of life of cancer patients at ORCI: training of health care professionals on assessment and management of spiritual issues, community education to correct misconceptions about causes and treatment of cancer, the importance of prompt seeking of medical care, and improvement in access to cancer treatment.
